# Seismicity characteristics based on the spatiotemporal ETAS model in North China from the perspective of different cut-off magnitude

**DOI:** 10.1371/journal.pone.0327295

**Published:** 2025-07-21

**Authors:** Jinmeng Bi, Fuyang Cao, Yong Ma

**Affiliations:** 1 Tianjin Earthquake Agency, Tianjin, China; 2 Institute of Geophysics, China Earthquake Administration, Beijing, China; Islamic Azad University Mashhad Branch, IRAN, ISLAMIC REPUBLIC OF

## Abstract

To explore the effect of the cut-off magnitude on the declustering of earthquake sequences, the calculation of seismicity parameters, the earthquake occurrence rate, and the hazard level of North China at present, we employed a stochastic declustering method based on the spatio-temporal ETAS model to decluster under different the cut-off magnitudes. And we have undertaken comprehensive research on the declustered distribution characteristics, seismicity parameters, and earthquake occurrence rates. The research results show that the selections of the cut-off magnitudes can lead to certain differences in the declustering results. As the cut-off magnitude increases, the declustering rate shows a certain downward trend, and the Poisson characteristics become more prominent. The stochastic declustering method does not significantly change the spatio-temporal statistical characteristics of seismicity parameters. Combining the analysis of the spatial distribution of the background earthquake occurrence rate, low *b*-value, and clustering rate in North China under different cut-off magnitudes, we found that regions including the intersection area of the Zhangjiakou-Bohai seismic belt and the Tanlu seismic belt, and the western part of the northern margin of the Ordos have relatively high seismic hazard. Some seismic belts, including the Zhangjiakou-Bohai seismic belt, exhibit a correlation among relatively high background seismicity, high crustal strain rate, and strong earthquakes. We can provide basic sequence data and technical support for the judgment of the dangerous state of moderate and strong earthquakes. This will further enhance our understanding of the laws of seismic activities in North China.

## Introduction

Reasonable declustering, calculation of seismicity parameters, and construction of earthquake models are three crucial aspects in seismic activity analysis. The selection of the cut-off magnitude can have a certain impact on the above issues [1–3]. The setting of the magnitude threshold cuts off the triggering relationship between earthquakes below the threshold and other earthquakes [4]. In addition, some aftershocks may be misclassified as enhanced background seismicity, leading to an overestimation of seismic hazards [5]. How to scientifically and reasonably classify clustered earthquakes is a crucial step in carrying out long-term, medium-term, and short-term earthquake forecasts and hazard assessments. The most common window-based declustering algorithms may cause excessive deletions during the post-earthquake stage [6]. Therefore, Zhuang et al. [7,8] proposed a stochastic declustering method based on the Epidemic-Type Aftershock Sequence (ETAS) model [9,10]. It can be used not only to analyze the background/clustering characteristics of earthquakes but also to effectively identify mainshocks, aftershocks, and swarms [11–13], which avoids the subjective selection of declustering parameters.

The seismicity parameter *b*-value represents the proportional distribution characteristics of different magnitudes [14]. However, it can vary with time, region, depth, and stress state [15], and is also significantly influenced by the cut-off magnitude [1]. The temporal variation characteristics of the *b*-value can better reveal the activity state of the earthquake sequence and reflect the stress changes. The spatial distribution of the *b*-value represents the cumulative level of regional stress. A low *b*-value (*b* < 0.7) can reflect the situation of fault locking or the presence of asperities [16]. Therefore, the time-space variation characteristics of the *b*-value are regarded as important clues for forecasting the potential large earthquakes.

Background seismicity serves as the foundation for earthquake forecasting and probabilistic seismic hazard analysis [17,18]. Among long-term earthquake occurrence rate models, Numerous methods have been proposed to evaluate the spatial occurrence rate function in time-independent models [19]. The kernel estimation method is the most widely employed, but in kernel estimation, due to the spatial distribution’s non-uniformity, it is challenging to use a fixed global bandwidth to represent the characteristics of the entire study area. To address this issue, Zhuang et al. [7] proposed an adaptive bandwidth kernel estimation method, which can better reflect the spatial non-uniformity of earthquakes and avoid over- or under-smoothing of regional earthquake activities. The research by Xiong et al. [20] indicate that the spatio-temporal ETAS model is effective in replicating the seismic activities, suggesting their feasibility in estimating future seismic hazards.

The North China Active Block Region is characterized by intense tectonic activities, well- developed faults and folds, and frequent seismic activities. Multiple strong earthquakes have occurred here, including the Hongdong *M*8.0 earthquake in 1303, the Huaxian *M*8.3 earthquake in 1556, and the Tangshan *M*7.8 earthquake that caused heavy losses. Moreover, North China has a complete historical earthquake catalog compared to other regions in China. Since the Zhangbei *M*6.2 earthquake in 1998, it has experienced a 27-year seismic quiet period, which has exceeded the longest previous quiet period, further increasing the urgency of a strong earthquake occurrence [21]. Many studies have been conducted on the long-term seismic hazards using various methods and paleoseismic or historical earthquake [16,20,22–24]. Regarding the *b*-value in the G-R relationship [14], Zheng and Zhou [25] and Guo [26] investigated its spatio-temporal or spatial variations in North China by an improved Bayesian method. The influence of different cut-off magnitudes on seismicity has not been systematically carried out in North China at present. We explored the effect of the cut-off magnitude on earthquake declustering, seismicity parameters, and earthqauke model construction, in order to provide a reference for reducing the seismic disaster risk in North China.

**Fig 1:**
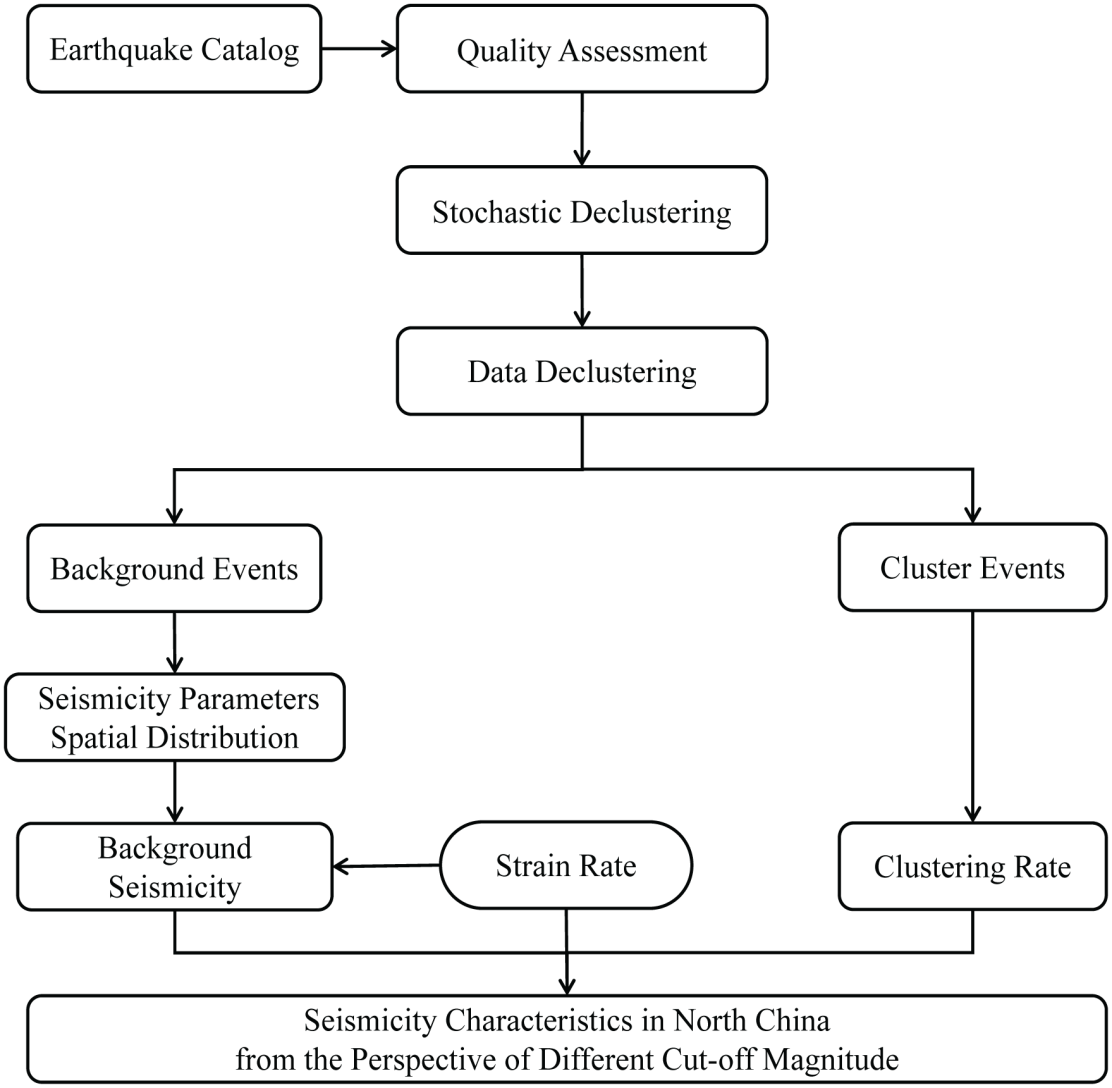
Workflow diagram of research methodology and components.

Previous studies have predominantly focused on analyzing background seismicity and clustered earthquake activity using a single cut-off magnitude [27,28]. While significant efforts have been directed toward methodological refinements [29], a systematic investigation into varying cut-off magnitudes remains notably absent. This gap is particularly critical given that the selection of cut-off magnitude substantially influences both earthquake sequence parameters [30] and forecasting efficacy [31]. To explore the impact of different cut-off magnitudes on seismicity parameters and background earthquake occurrence rate, the research flowchart is illustrated in Fig 1, we processed the earthquake catalog of North China using the stochastic declustering method. And we analyzed the spatio-temporal distribution of the declustering results under different cut-off magnitudes, as well as their effects on seismicity parameters and earthquake occurrence rates. Moreover, we evaluated the current hazard level in North China by combining the background occurrence rate, *b*-value, clustering rate, and strain rate, etc.

## Data and methods

**Fig 2:**
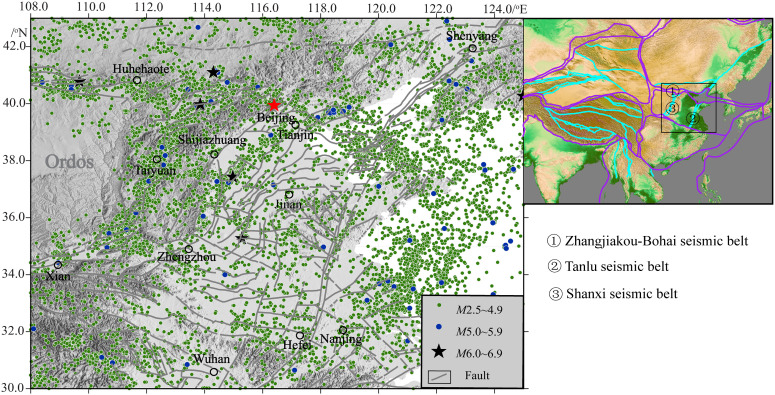
Distribution map of seismic activities in North China. The gray curves in the base map represent active tectonics.

We used the the earthquake catalog of “National Unified Official Cataloging” provided by the China Earthquake Networks Center [National Earthquake Cataloging System, http://10.5.160.18/console/exit.action]. Since 1980, a total of 17,988 earthquakes with *M*_L_ ≥ 2.5 have occurred in North China. Among them, there are 5,782 earthquakes with magnitudes ranging from 3.0 to 3.9, 717 earthquakes with magnitudes from 4.0 to 4.9, 100 earthquakes with magnitudes from 5.0 to 5.9, and 10 earthquakes with magnitudes from 6.0 to 6.9. This includes the Zhangbei *M*6.2 earthquake in Hebei province on January 10, 1998, and the Pingyuan *M*5.5 earthquake in Shandong province on August 6, 2023, which have had a huge social impact. Fig 2 shows the distribution characteristics of earthquakes with *M*_L_ ≥ 2.5 in North China (30.0–43.0°N, 108.0–125.0°E) since 1980. From the spatial distribution of earthquakes, it is evident that earthquakes exhibit a distinct clustering pattern along seismic belts, with particularly notable strong earthquakes consistently aligned along these zones.

**Fig 3:**
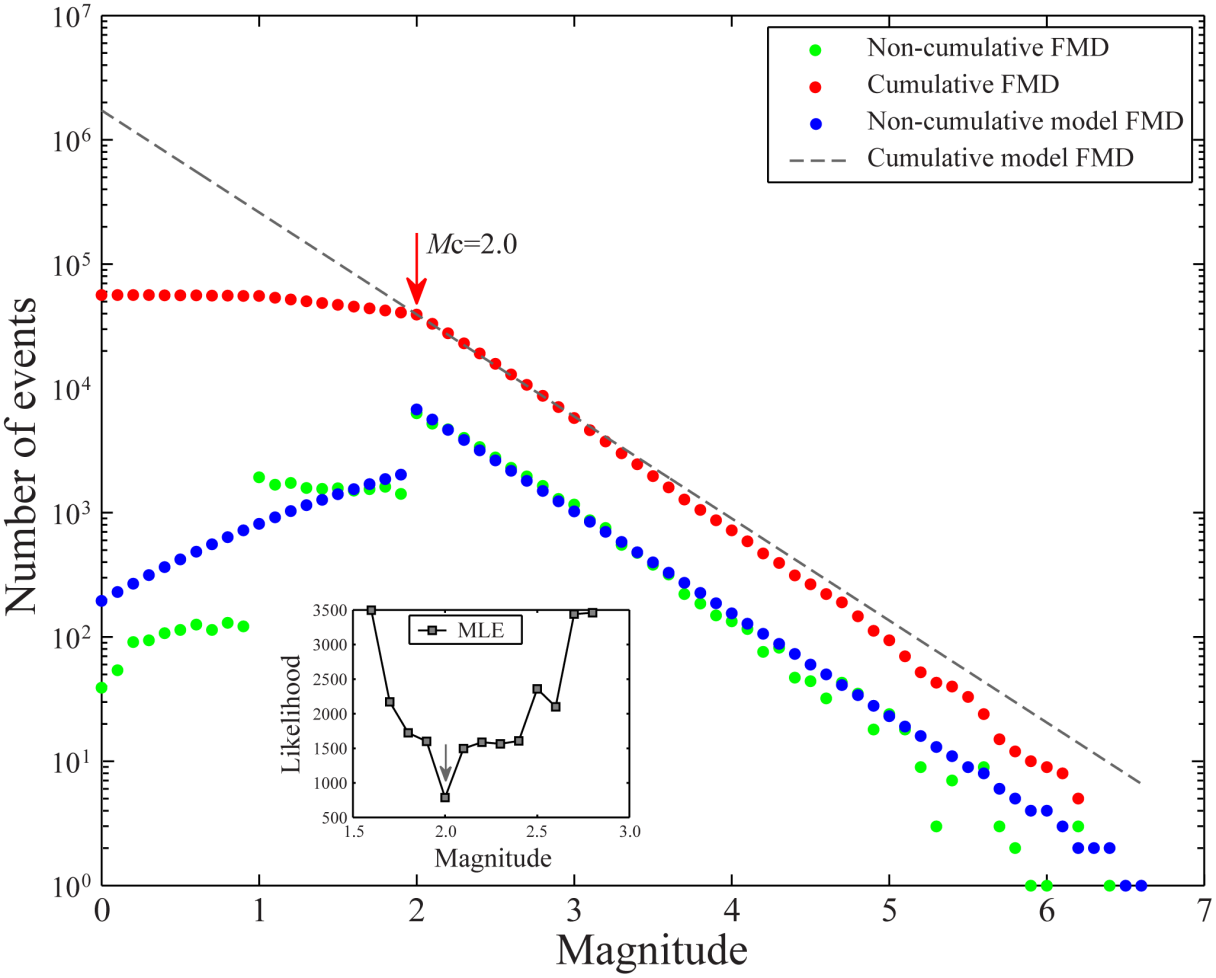
Magnitude-frequency distribution and magnitude completeness analysis results in North China.

Bi et al. [21] conducted a quality assessment of the earthquake catalog in North China since 1970 using a combination of qualitative and quantitative methods, such as the magnitude-rank method [32,33], the maximum curvature method, and the goodness-of-fit method [34]. To mitigate this impact and ensure the reliability of the spatio-temporal ETAS model parameter estimation, the Entire-Magnitude-Range (EMR) method [1] was used to evaluate the overall *M*_c_ in North China from January 1, 1980, to December 31, 2023. The corresponding magnitude-frequency distribution is shown in Fig 3. In the sub-figures of Fig 3, the variation of the maximum likelihood value of the estimated *M*_c_ between *M*1.5 and *M*3.0 is also presented. For the sake of convenience, the values of Maximum Likelihood Estimation (MLE) are presented with opposite signs. The maximum likelihood value is the smallest when *M*_c_ = 2.0, which corresponds to the minimum completeness magnitude. Combined with the results of the dynamic monitoring ability assessment based on qualitative and quantitative methods [21], and taking into account the minor oscillatory changes in the minimum completeness magnitude in the early stage, *M*_c_ = *M*_L_2.5 was selected as the minimum completeness magnitude for the North China seismic region to conduct subsequent declustering processing and comparative analysis.

Based on Omori’s law, Ogata [9] developed the ETAS model founded on the theory of point branching processes. This model assumes that each earthquake independently triggers “aftershocks” according to certain probability rules. Ogata [10] proposed that the earthquake occurrence rate of the spatio-temporal ETAS model can be expressed as:


$$\lambda (t,x,y)=s(m)[\mu (x,y)+\sum_{i:t_{i}<t}\kappa (m_{i})g(t-t_{i})f(x-x_{i},y-y_{i};m_{i})]$$ (1)

Here, *μ*(x, y) represents the background seismicity intensity, which is a spatial function related to location; *s*(*m*) is the probability density function of earthquakes and can be expressed as *s*(m)=*β*exp[-*β*(*m*-*m*_c_)], where *m* ≥ *m*_c_. *β* is a constant, expressed as *β* = *b* ln10. The value of *b* can be obtained through the Gutenberg-Richter (G-R) relationship, and *m*_c_ is the cut-off magnitude; *κ*(*m*) represents the expected number of aftershocks triggered by an earthquake of magnitude *m* and can be expressed as $\kappa (m)=Ae^{\alpha (m-m_{c})},m\geq m_{c}$; *g*(t) and *f*(x, y; m) are the time and spatial probability density functions of aftershocks respectively, and are expressed as $g(t)=\frac{p-1}{c}{\left(1+\frac{t}{c}\right)}^{-p},t>0$ and $f(x,y;m)=\frac{q-1}{\pi De^{\gamma (m-m_{c})}}{\left(1+\frac{x^{2}+y^{2}}{De^{\gamma (m-m_{c})}}\right)}^{-q}$ respectively.

Each parameter θ=(μ,A,c,α,p,D,q,γ) is estimated using the maximum likelihood estimation.


$$\log L(\theta )=\sum_{j:(t_{j},x_{j},y_{j})\in S\times [T_{1},T_{2}]}\log\lambda (t_{j},x_{j},y_{j})-\int \int_{S}\int_{T_{1}}^{T_{2}}\lambda (t,x,y)dtdxdy$$ (2)

The relative contribution of earthquake *i* to the earthquake occurrence rate at the location (*t*_j,_ *x*_j_, *y*_j_) of earthquake *j* that occurs later, or the probability of triggering earthquake *j*, can be expressed as [7,35]:


$$\rho_{ij}=\varsigma_{i}(t_{j},x_{j},y_{j})/\lambda (t_{j},x_{j},y_{j}),j>i,$$ (3)

and $\varsigma_{i}(t,x,y)=\kappa (m_{i})g(t-t_{i})f(x-x_{i},y-y_{i}|m_{i})$_,_

The probability that earthquake *j* is a background earthquake is:


$$\phi_{j}=\frac{\mu (x_{j},y_{j})}{\lambda (t_{j},x_{j},y_{j})}.$$ (4)

The probability that earthquake *j* is triggered by a previous earthquake, i.e., the clustering probability, can be expressed as:


$$\rho_{j}=1-\phi_{j}=\sum_{i}\rho_{ij}$$ (5)

The probability of background earthquakes is used to estimate the intensity of background seismic activity $\hat{\mu }(x,y)$ by the kernel function method.


$$\hat{\mu }(x,y)=\frac{1}{T}\sum_{i}\phi_{i}Z_{h_{i}}(x-x_{i},y-y_{i})$$ (6)

Among them, *T* represents the time scale, and *Z*_hj_ is the value of the Gaussian kernel function with a bandwidth of *h*_j_. The bandwidth variable *h*_*j*_ is the distance from earthquake *j* to the *n*_p_-th nearest earthquake.

The kernel function estimation of the overall earthquake occurrence rate:


$$\hat{m}(x,y)=\frac{1}{T}\sum_{i}Z_{h_{i}}(x-x_{i},y-y_{i})$$ (7)

The estimation of the clustered earthquake occurrence rate can be expressed as:


$$\overset{\wedge }{C}(x,y)=\frac{1}{T}\sum_{i}\rho_{i}Z_{h_{i}}(x-x_{i},y-y_{i})$$ (8)

The actually occurring earthquake catalog can be expressed as $\left\{(t_{i},x_{i},y_{i},m_{i}):i=1,2,...,N\right\}$. Assuming that the earthquake intensity function is known as u(x,y), the activity intensity of background earthquakes can be expressed as μ(x,y)=vu(x,y). Here, v is an unknown parameter greater than 0, and each parameter in formula (1) can be estimated by the maximum likelihood method. After obtaining the background earthquake probability of each earthquake, recalculate and repeat the iteration until the result converges. Finally, obtain the background earthquake activity intensity and the model parameters {*μ*, *A*, *α*, *p*, *q*, *D*, *γ*, *c*}.

## Distribution characteristics of earthquakes

The spatio-temporal ETAS model parameters under different cut-off magnitudes in North China from January 1, 1980, to December 31, 2023, were estimated using the maximum likelihood method. To compare the differences in the output parameters, the same initial input parameters were selected. The initial values and maximum likelihood estimation results of the model parameters are presented in Table 1. Moreover, the stochastic declustering method was employed to relatively scientifically separate the earthquake sequences in North China. As a result, the probability *φ* of each earthquake being an isolated/background earthquake and the probability *ρ* of any earthquake being triggered by a previously occurring “parent earthquake” were obtained. Earthquakes with a background probability *φ* ≥ 0.95 were used to form the background earthquake catalog (declustered catalog), and the remaining earthquakes constituted the clustered earthquake catalog.

**Table 1:** Parameters from fitting the space-time ETAS model to seismicity in North China.

Parameter		*ν*	*A*	*c*	*α*	*p*	_*D*_	_*q*_	*γ*
Initial parameter		1.2141	0.7604	0.0021	1.2360	1.0152	0.0001	1.1980	1.3716
Output parameter	*M*2.5	1.0242	0.5432	0.0438	0.4440	1.0324	0.0001	1.5578	0.0043
	*M*3.0	0.6994	0.5275	0.0214	0.1482	1.0589	0.0001	1.0142	0.0010
	*M*3.5	0.7200	1.0221	0.0005	0.9193	1.0119	0.0001	1.0047	0.0001
	*M*4.0	0.7100	0.4292	0.0005	1.8720	1.0169	0.0001	1.0026	0.0001
	*M*4.5	0.7200	1.0221	0.0005	0.9193	1.0119	0.0001	1.0047	0.0001
	*M*5.0	0.7716	0.2645	0.0204	1.1454	1.0322	0.0001	1.0059	0.0082

**Fig 4:**
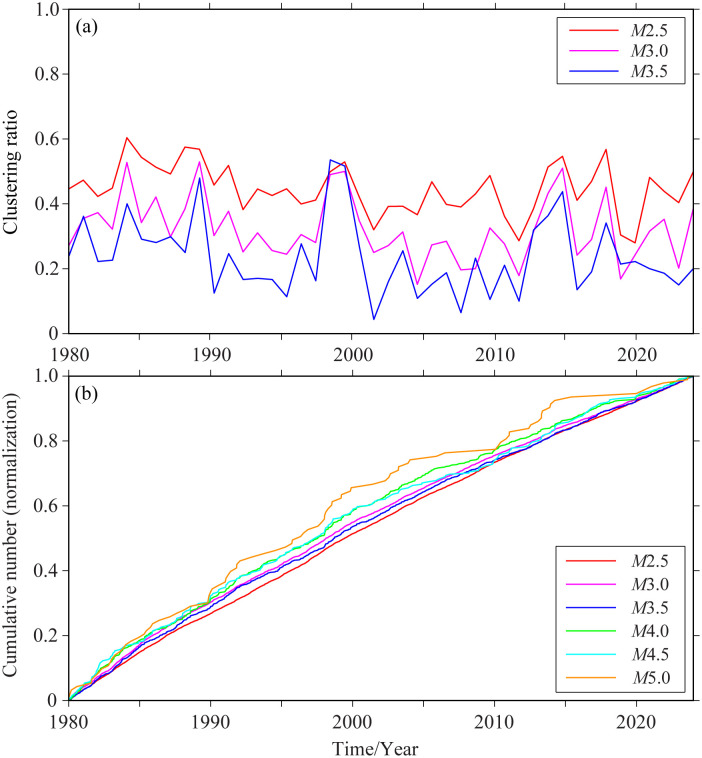
Declustering results under different cut-off magnitudes. (a) Variation of declustering rate over time (b) Normalized results of cumulative background earthquakes.

Here, the declustering rate is defined as the ratio of the number of removed earthquakes (background probability *φ* < 0.95) to the total number of earthquakes. Table 2 presents the declustering results under different cut-off magnitudes. Fig 4a shows the variations of the declustering rate over time for three cut-off magnitude levels: 2.5, 3.0, and 3.5. With the cut-off magnitude increases, the declustering rate shows a downward trend, which also implies that stronger earthquakes have a relatively high probability of being regarded as background earthquakes. Moreover, due to the reduction in the number of samples, relatively large fluctuations occur. Temporally, the declustering rate exhibits similar fluctuating patterns. That is, under different cut-off magnitudes, the changing trends of declustering are basically consistent.

**Table 2:** Calculation results of seismic activity parameters in study area.

	Declustering results			Seismic activity parameters						Poisson test	
	Total earthquakes	Background earthquakes	Declustering rate	*a*_all_	*b*_all_	*a*_bg_	*b*_bg_	*a*_cluster_	*b*_cluster_	K-S	Cv
*M*2.5	17988	9771	45.68%	6.45	0.88 ± 0.01	6.25	0.90 ± 0.01	6.05	0.85 ± 0.01	0.05	1.04
*M*3.0	6609	4361	34.01%	6.56	0.91 ± 0.01	6.41	0.92 ± 0.01	6.04	0.90 ± 0.02	0.10	1.05
*M*3.5	2259	1668	26.16%	6.45	0.88 ± 0.02	6.39	0.91 ± 0.02	5.67	0.84 ± 0.03	0.21	1.02
*M*4.0	827	603	27.09%	6.47	0.89 ± 0.03	6.31	0.88 ± 0.03	5.95	0.90 ± 0.06	0.43	1.09
*M*4.5	307	250	18.57%	6.60	0.91 ± 0.04	6.44	0.90 ± 0.05	6.24	1.00 ± 0.12	0.20	1.07
*M*5.0	110	88	20.00%	7.22	1.04 ± 0.09	7.17	1.04 ± 0.10	6.37	1.01 ± 0.18	0.01	1.37

The periods with a relatively high declustering rate are mainly associated with earthquakes such as the Zhangbei *M*6.2 earthquake on January 10, 1998, the Rushan earthquake swarm from October 1, 2013, to February 13, 2016, and the Changdao earthquake swarm from February 14, 2017, to February 28, 2018. Prominent earthquakes and regional earthquake swarm activities can cause a sharp increase in the declustering rate. Overall, the selection of the cut-off magnitude does not affect the changing trend of the declustering rate of earthquake sequences over time.

To further compare the variations of background earthquakes under different cut-off magnitudes, the cumulative number of background earthquakes over time was selected, and the results were normalized. As shown in Fig 4b, the theoretical and actual curves of background seismicity over time are presented. If the model fits well with the seismicity, or the background occurrence rate is a constant function of time, when the slope decreases, it indicates a quiet period in the background seismic activity; while if the slope increases, it is called an active period in the background seismicity [36]. Overall, the background earthquakes basically show a certain constant change under different cut-off magnitudes. As the cut-off magnitude increases and the number of samples decreases, the fluctuation range is relatively large, showing periodic quiet and active periods. For cut-off magnitudes of *M* ≤ 4.0 and below, the background earthquakes exhibit changes consistent with the Poisson distribution characteristic curve. For cut-off magnitudes of 4.5 and 5.0, there is an alternating phenomenon of quiet and active periods, and currently, it is in a quiet period.

The declustering results of earthquake catalogs are mainly used in the construction of earthquake forecasting models and probabilistic seismic hazard analysis. The assumption for constructing the seismicity model in probabilistic seismic hazard analysis is that the occurrence of earthquakes follows a Poisson distribution. Therefore, to verify whether the declustering results under different cut-off magnitudes conform to the Poisson distribution, the declustering results were examined from multiple perspectives. To assess the declustering effectiveness, first, the Kolmogorov-Smirnov (K-S) hypothesis testing method was employed to conduct a Poisson test for the temporal stationarity of background earthquakes obtained from the stochastic declustering algorithm. This method is primarily used to check whether the actual distribution of sample data matches a specified theoretical distribution. Second, the coefficient of variation of the time intervals between earthquakes for each catalog was calculated. The coefficient of variation is defined as the ratio of the standard deviation of the time intervals between earthquakes to the average of the time intervals between earthquakes in the catalog and can be used to determine whether the earthquake catalog follows a Poisson distribution in time. When the catalog approximates a Poisson distribution, the coefficient of variation (Cv) approaches 1 [37]. The calculation results are presented in Table 2.

Based on this, the declustering scenarios under different cut-off magnitudes were analyzed. The inspection results show certain differences. The earthquake catalogs obtained under most cut-off magnitudes follow a Poisson distribution. Except for the cut-off magnitude of 5.0, where the number of samples is relatively small and does not meet the Poisson distribution, the other inspection results satisfy the characteristics of the Poisson distribution. Moreover, as the cut-off magnitude increases, the Poisson characteristics become more prominent. However, the reduction in the number of samples is also an important factor affecting the characteristics of the Poisson distribution.

## Distribution characteristics of seismicity parameters

### The overall distribution characteristics

The *b*-value is related to factors such as the stress state of crustal rocks and the heterogeneity of the medium. Currently, the commonly used methods for calculating the *b*-value mainly include the maximum likelihood method [38] and the least-squares method. Among them, the maximum likelihood method is relatively simple to calculate and is less affected by individual large earthquakes. It calculates the average with the same weight for earthquakes in all magnitude bins, which can, to a certain extent, eliminate the sudden change in the *b*-value caused by the occurrence of individual significant earthquakes. The maximum likelihood method is a commonly used point-estimation method. Its essence is to take the parameter value that maximizes the probability of the observed earthquake samples as the estimate of the unknown parameter.

**Fig 5:**
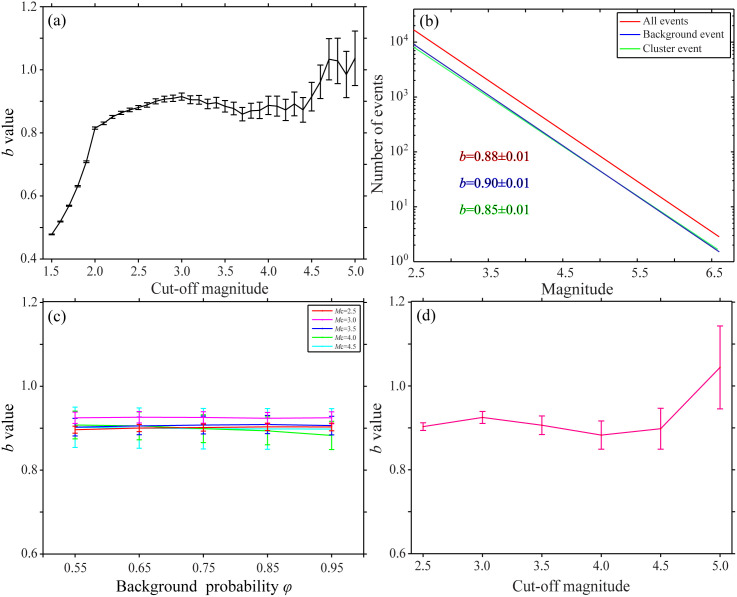
The relationship between threshold setting and seismicity parameters. (a) Plot of the variation of *b*-value with the cut-off magnitude (b) Example of calculating the *b*-value by the maximum likelihood method (c) Plot of the relationship between the *b*-value and the background probability (d) Plot of the relationship between the *b*-value of background earthquakes and the cut-off magnitude.

Generally speaking, a higher *b*-value indicates a higher proportion of small earthquakes relative to large earthquakes, suggesting that the crustal medium is relatively fragmented and the stress distribution is relatively uniform. Conversely, when the *b*-value is low, it means that there are relatively few small earthquakes, which may imply that the crustal medium is relatively intact and the stress is concentrated on a few major fault zones. To explore the differences in the *b*-value under different cut-off magnitudes, which was calculated using the maximum likelihood method. As shown in Fig 5a, when the cut-off magnitude is small, as the cut-off magnitude increases, the *b*-value also increases. This may be due to the lack of small earthquakes. When the cut-off magnitude increases to a certain range, the *b*-value fluctuates within a certain range. With the continuous increase of the cut-off magnitude, the *b*-value will have a relatively large error.

To further investigate the influence of earthquake declustering on the *b*-value under different cut-off magnitudes, the *b*-values of all earthquakes, background earthquakes, and clustered earthquakes were calculated using the maximum likelihood method as shown in Table 2 and Fig 5b. The differences among the three are generally not significant. Contrary to the traditional view that the *b*-value of the mainshock should be lower than that of aftershocks, the *b*-value of background earthquakes is higher than that of clustered earthquakes in the low-magnitude range (2.5–3.5) in study area. This may be because the background earthquake corresponds to the first earthquake in the sequence rather than the largest earthquake, and the *b*-values of background earthquakes and mainshocks are determined by the average magnitudes of the first earthquake and the largest earthquake in the sequence, respectively. Specifically, the larger the average magnitude, the smaller the *b*-value. This phenomenon, where the average magnitude of background earthquakes is lower than that of the mainshock, also indicates that the first earthquake in the earthquake sequence in study area tends to be a small earthquake, that is, a foreshock in the traditional sense.

From the perspective of the *a-*value, which reflects the overall level of seismicity, for the same magnitude range, the *a*-value for total earthquakes is greater than that for background earthquakes, which is greater than that for clustered earthquakes. This also shows that the overall level of background seismicity is higher than that of clustered seismicity. Moreover, different cut-off magnitudes have an insignificant impact on the *a*-value. In addition, the number of earthquakes is a key factor affecting the stability of the *b*-value. When the number of earthquakes (sample size) is small, the error is relatively large. When the sample size reaches a certain scale, as the sample size increases, the *b*-value of seismic activity parameters will gradually tend to be stable, and the influence of the cut-off magnitude can be basically ignored.

To ensure the objectivity of the *b*-value, we further analyzed the variation characteristics of the *b*-value under different background probabilities. We carried out tests for different background probability thresholds. We selected five parameters, 0.95, 0.85, 0.75, 0.65, and 0.55 respectively, for calculation. The results are shown in Fig 5c and Table 2. Under the same magnitude threshold, the setting of the background probability threshold has little correlation with the variation of the *b*-value. The *b*-value basically fluctuates within a certain range. This also reveals that the stochastic declustering algorithm has a relatively small impact on seismicity parameters, and the declustering results will not significantly change the statistical characteristics. During the calculation process, on the premise of ensuring the earthquake catalog completeness and the earthquake number, we explored the declustering effects under six different magnitude thresholds of 2.5, 3.0, 3.5, 4.0, 4.5, and 5.0 and their impacts on subsequent seismicity parameters. The results are shown in Fig 5d and Table 2.

As the magnitude threshold increases, the declustering rate shows a downward trend. This result shows certain differences from the analysis conclusions regarding the relationship between the *b*-value and the magnitude threshold using declustering algorithms such as Gardner and Knopoff and Reasenberg [39–41]. That is, the *b*-value does not change significantly with the variation of the magnitude threshold. It was found that the *b*-value generally does not change much with the variation of the cut-off magnitude. Due to the decrease in the number of samples, the error shows a certain increasing trend. When *M*_c_ is taken as 5.0, the *b*-value is relatively large and the error is also relatively large. Analyzing the impacts of different threshold conditions on the declustering rate and the seismicity parameters *a*-value and *b*-value plays a crucial role in accurately grasping the characteristics of seismic activity and improving the accuracy and reliability of probabilistic seismic hazard analysis. Therefore, the proportion of small and large earthquakes deleted by the stochastic declustering method is relatively stable. The selection of the cut-off magnitude and the background probability threshold do not significantly change the statistical characteristics of seismic activity parameters.

### The spatial distribution characteristics of seismic activity parameters

The spatial distribution of the *b*-value represents the cumulative level of regional stress and it is an intuitive manifestation of the changes in site stress conditions. The *b*-value is mainly related to the stress conditions of the crust, the degree of fault creep, and the complexity of the fault trace. Therefore, the heterogeneous distribution and evolution law of the spatially distributed *b*-value are regarded as important clues for forecasting the location and magnitude of potential major earthquakes.

**Fig 6:**
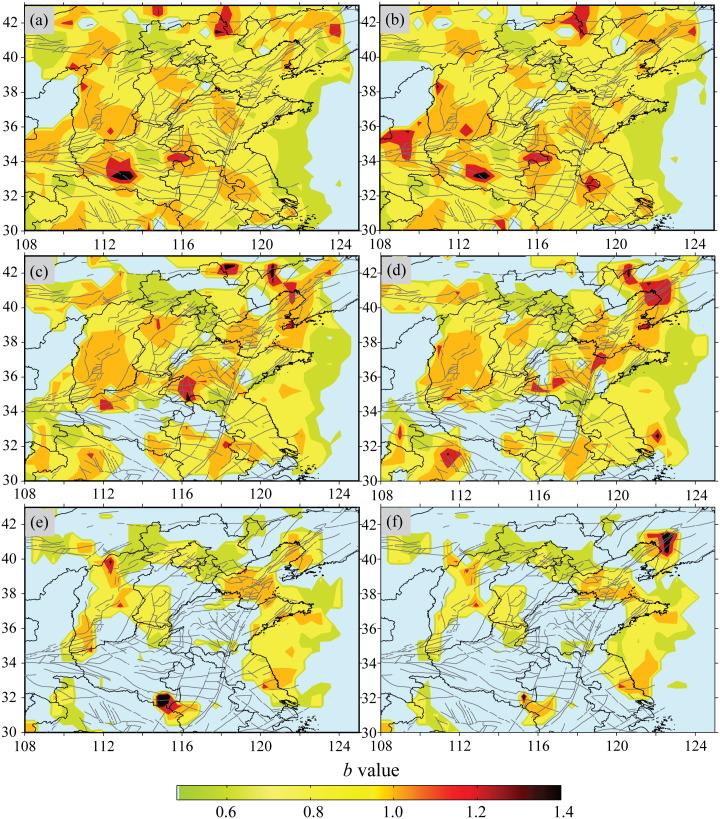
Distribution characteristics of *b*-values for all earthquakes and different cut-off magnitudes. (a) All earthquakes with *M* ≥ 2.5. (b) Background earthquakes with *M* ≥ 2.5. (c) All earthquakes with *M* ≥ 3.0. (d) Background earthquakes with *M* ≥ 3.0. (e) All earthquakes with *M* ≥ 3.5. (f) Background earthquakes with *M* ≥ 3.5.

Due to the differences in regional activity characteristics, the declustering results of different declustering algorithms show certain variations. To reveal the regional activity characteristics under different cut-off magnitudes, Fig 6 presents the spatial scanning results of the *b*-value for all earthquakes and different cut-off magnitudes. The cut-off magnitude has varying degrees of influence on the spatial distribution of the *b*-value. However, the spatial distribution of the *b*-value under different cut-off magnitudes shares certain commonalities. That is, there are relatively low *b*-value anomalies in the western part of the northern margin of the Ordos and the Zhangjiakou-Bohai seismic belt. As the cut-off magnitude increases, the low *b*-value anomaly area shows a certain contraction. Firstly, this is related to the reduction in the number of earthquake samples, resulting in a smaller number of samples involved in the calculation. Secondly, major earthquakes tend to occur more aggregately. This is basically consistent with the results obtained by Xiong et al. [20] based on the ETAS model, HIST-ETAS model, and *b*-value using the earthquake catalog from 1970 to 2016.

From the perspective of the overall spatial distribution, there are significant low *b*-value anomalies in regions such as the western part of the northern margin of the Ordos and the Zhangjiakou-Bohia sismic belt. When compared with the *b*-values of North China calculated by Guo [26] using the hierarchical ETAS model, there is some consistency. In the western section of the Zhangjiakou-Bohai seismic belt, the presence of a low *b*-value anomaly might be related to the significant absence of aftershocks following the Zhangbei *M*6.2 earthquake on January 10, 1998. In the intersection area of the Zhangjiakou-Bohai seismic belt and the Tanlu seismic belt, and the western part of the northern margin of the Ordos, there are relatively large-scale low *b*-value anomalies. The stress state in these areas is at a high level, indicating a high risk of strong earthquakes in the future.

However, accurately measuring the absolute stress level in a specific region is extremely challenging. To better understand the *b*-value, it can be combined with the image of Coulomb stress changes, achieving a mutual verification effect. Coulomb failure stress change can provide valuable stress information, which is conducive to understanding the characteristics of regional seismic activities. The Coulomb failure stress change generated by strong earthquakes can either promote or inhibit subsequent seismic activities. Shen et al. [42] presented the evolutionary process of the cumulative Coulomb failure stress change in North China since 700 AD, which was caused by long-term tectonic loading and seismic faulting. Both the western part of the northern margin of the Ordos and the Zhangjiakou-Bohia sismic belt are regions where the current Coulomb failure stress has increased significantly. This shows the consistency between the *b*-value and the Coulomb stress changes of strong earthquakes.

### Variation characteristics of parameters of typical tectonic belts

The Zhangjiakou-Bohai seismic belt is currently a key area exhibiting a low *b*-value, and we have carried out a targeted study on the characteristics of its sequence changes. To reveal the temporal variation characteristics of the *b*-value, we utilized the *b*-value calculation methods based on data-driven and the OK1993 model [16,43] to analyze the seismic sequences in the Zhangjiakou-Bohai seismic belt since 2010. The main calculation steps of this method include the selection of the magnitude-frequency distribution function, the random division of the time axis, the selection of the best model, and the calculation of the median value of the *b*-value set. In addition, Jiang et al. [44] found that the *b*-value calculation results obtained under different combinations of the number of segmentation periods (*n* + 1) and the number of multiple random segmentations *w* are relatively close, indicating the stability of this method. Therefore, for the main seismic belts in study area, the BIC value distribution and the temporal variation of the *b*-value were obtained with the number of segmentation periods *n* + 1={2, 3,... 11} and *w* = 100.

**Fig 7:**
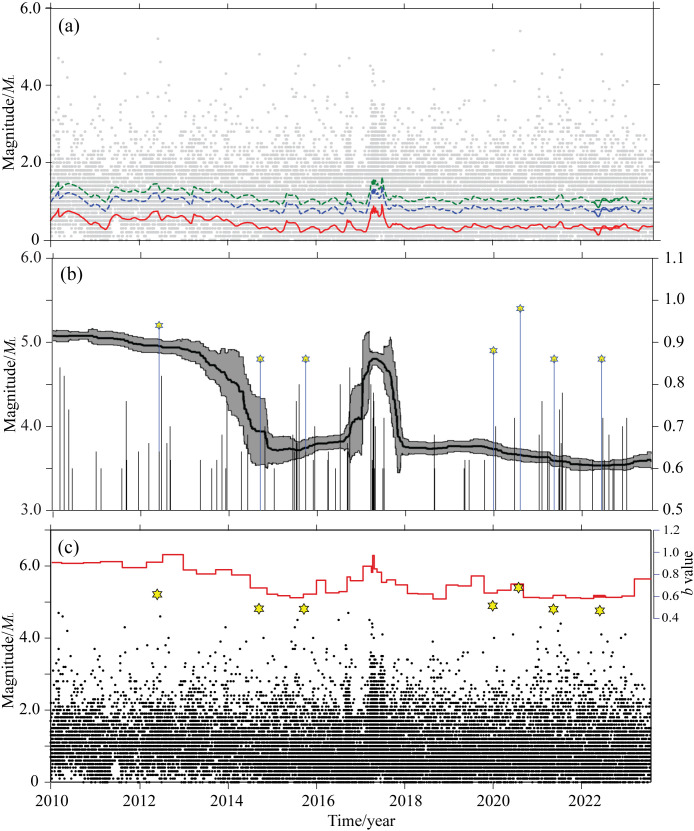
Variation characteristics of the *b*-value in the Zhangjiakou-Bohai seismic belt. (a) Minimum completeness magnitude analysis of the earthquake sequence. (b) The *b*-value calculated using the data-driven method and the OK1993 model. (c) The *b*-value calculated by the fixed-window method.

To demonstrate the variation of the minimum completeness magnitude of seismic sequences in the Zhangjiakou-Bohai seismic belt, Fig 7a presents the median value of the set of *μ* calculated based on the OK1993 model, as well as the results of *μ* + 2*σ* and *μ* + 3*σ*. The temporal parameter changes of the *b*-value calculated based on data-driven and the OK1993 model are shown in Fig 7b. The *b*-value results calculated based on the data-driven method and the OK1993 model are presented. It can be observed that during the occurrence of earthquake sequences in the Zhangjiakou-Bohai seismic belt, the *b*-value is generally lower than 1.0. The *b*-value experiences certain fluctuations, and these fluctuations are accompanied by relatively large Median Absolute Deviation (MAD) values. From January 2010 to April 2013, the *b*-value showed a slight downward trend, gradually decreasing from 0.92 to 0.87. Subsequently, it exhibited a rapid downward trend. From May 2013 to September 2014, the *b*-value dropped from 0.87 to 0.65. Between October 2014 and July 2016, the *b*-value of the sequence parameters fluctuated slightly at a low level, rising from 0.65 to 0.67. Then, it showed a rapid increase. From July 2016 to February 2017, the *b*-value rose from 0.67 to 0.86. From July to November 2017, it showed a rapid decline, dropping from 0.86 to 0.65, and then continued to fluctuate within the range of low *b*-values. Overall, 6 out of 7 earthquakes with *M* ≥ 4.5 occurred during the low *b*-value phases.

To compare the characteristics of parameter variations, the *b*-value was also calculated along the time axis with a step size and window length of 500 earthquakes. The corresponding results are shown in Fig 7c. It was found that the *b*-value of the fixed-time window exhibited multiple fluctuations, which may better reflect the activity characteristics of the sequence. The overall *b*-value ranged from 0.58 to 0.97, with an average of 0.73 ± 0.10. Before the occurrence of the seven earthquakes with *M* ≥ 4.5 in the Zhangjiakou-Bohai seismic belt, there were low *b*-value anomalies to varying degrees, which reflected the stress accumulation before the occurrence of significant earthquakes. The phenomenon of a decrease in the *b*-value occurred before the occurrence of most significant earthquakes. The results obtained by the two *b*-value calculation methods adopted in this study show strong consistency. There were low *b*-value anomalies to varying degrees before the occurrence of moderate-to-strong earthquakes, which also indicates that the stress level was relatively high before the occurrence of moderate-to-strong earthquakes.

## Assessment of seismic activity status

Background seismic activity results from a relatively slow and long-term tectonic stress loading process. It is associated with long-term tectonic phenomena and can effectively reflect the loading rate of the gravitational field. In contrast, clustered seismic activity is mainly attributed to stress changes induced by previous earthquakes. It is primarily caused by significant earthquakes and subsequent seismic activities, demonstrating the clustering seismicity in spatial distribution. A high clustering rate is a major manifestation of the clustering seismicity. It indicates the presence of specific tectonic activities or stress-concentrated areas in the region. Along the fault zones near plate boundaries, a high clustering rate may suggest that the fault zone is undergoing a strong stress adjustment process.

### Background seismic activity

**Fig 8:**
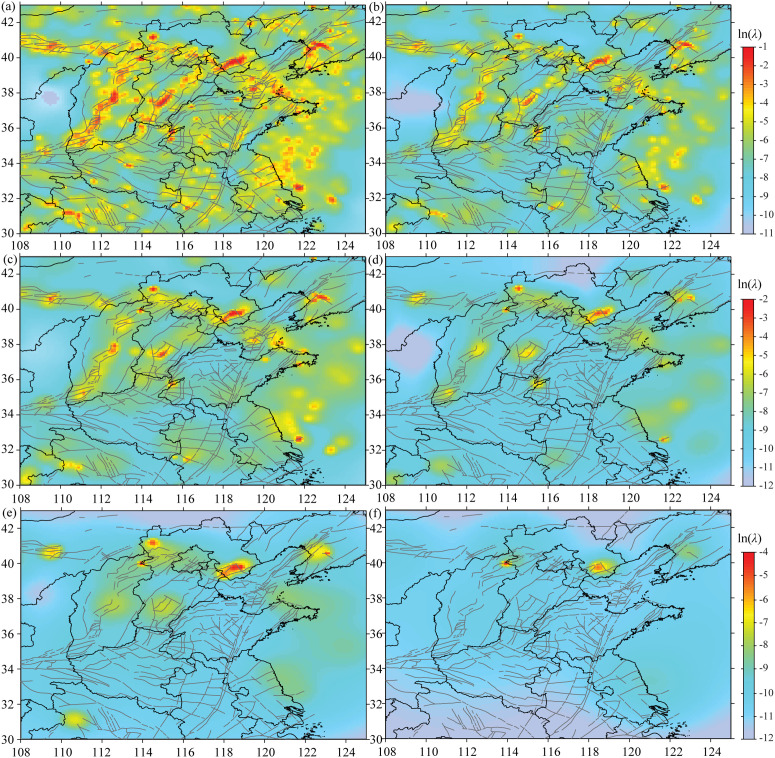
Distribution characteristics of background seismicity under different cut-off magnitude. (a) *M*2.5, (b) *M*3.0, (c) *M*3.5, (d) *M*4.0, (e) *M*4.5, (f) *M*5.0.

The background occurrence rate reflects the basic activity level in a specific region under geological tectonic stress, without the influence of obvious short-term triggering factors. Background earthquakes are stationary in time but non-uniform in space, which may be affected by the changes in the regional tectonic stress field. Fig 8 shows the spatio-temporal variation characteristics of background seismicity under different cut-off magnitude Spatially, the distribution of seismicity in study area is largely composed of major fault zones. Most of the regions with a relatively high seismicity rate are located on the main seismic belts in study area, mainly in the Zhangjiakou-Bohai seismic belt, the Shanxi seismic belt, the North China Plain seismic belt, and the Yellow Sea area. There are several local high-value areas of seismic activity rate in other regions, which are caused by several significant earthquake sequences or earthquake swarms.

Under different cut-off magnitude, the background seismic occurrence rates exhibit similar distribution characteristics, that is, they are mainly distributed along the fault zones. However, as the cut-off magnitude increases and the number of samples decreases, the background occurrence rate shows an obvious downward trend, and the areas with high occurrence rates tend to shrink. Regions such as the central and southern segments of the Shanxi seismic belt, the intersection area of the Tanlu seismic belt and the Zhangjiakou-Bohai seismic belt, the central and southern segments of the North China Plain seismic belt, and the southwestern of the Yellow Sea are among the relevant high-risk areas.

### Clustering rate analysis

The seismic clustering rate method mainly identifies fault stress-locked segments or asperities through low seismic clustering rates in space [36,45]. This method is conducive to revealing the spatial inhomogeneity of seismicity and reflects the differences in crustal structure and stress states in different regions. By removing the background seismicity component, the clustering rate contains most of the irregular components of the total seismicity. Its spatial variation reveals a more complex pattern than the seismicity rate. Areas with a low clustering ratio indicate that earthquakes are less likely to occur within the region. However, once a major earthquake occurs, it is highly likely to be accompanied by a large number of aftershocks. The asperities (locked segments) of faults are located in areas with low clustering rates but close to regions with high backgrounds and high clustering rates [45], which deserve particular attention in the future.

**Fig 9:**
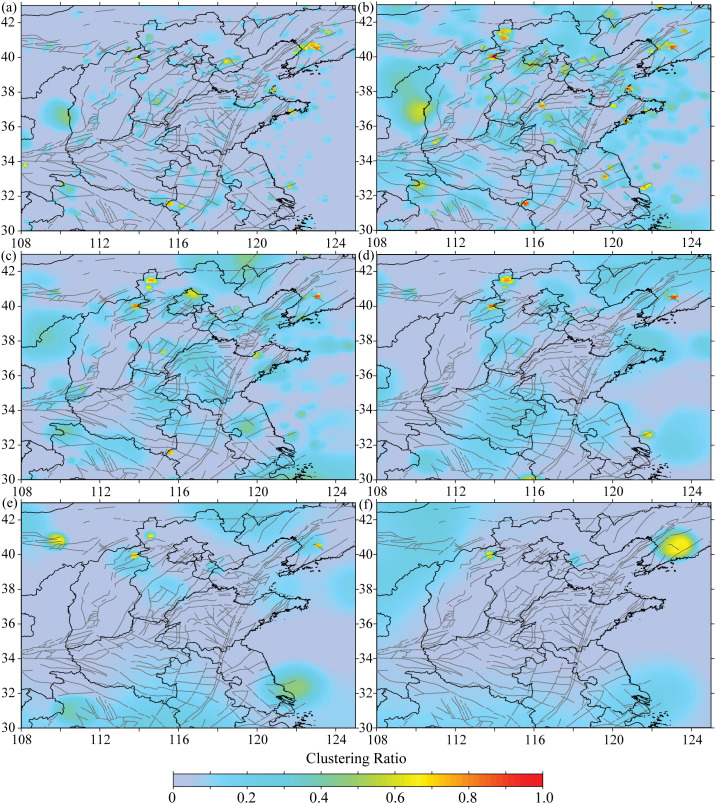
The spatial distribution of the clustering rate under different cut-off magnitude. (a) *M*2.5, (b) *M*3.0, (c) *M*3.5, (d) *M*4.0, (e) *M*4.5, (f) *M*5.0.

Under different cut-off magnitude, the spatial distribution of the clustering rate exhibits certain differences in Fig 9. As the cut-off magnitude increases, due to the reduction in the number of samples, the spatial extent of high clustering rates shows a certain shrinking trend. Constrained by the seismic spatial distribution and the number of samples in study area, the high clustering rates show a punctate distribution over small areas. By combining the locations of locked segments, potential hazardous areas for moderate to strong earthquakes can be identified. These areas include the junction region of Shanxi, Hebei, and Inner Mongolia, the middle-eastern section of the Zhangjiakou-Bohai seismic belt, the middle-northern section of the Tanlu seismic belt, and the southwestern of the Yellow Sea. These regions may be the areas that deserve particular attention in study area in the future.

### Relationship between the seismicity characteristics and strain rate

Behind seismic activity lies the influence of physical mechanisms. The statistical characteristics revealed by numerous observations often mirror the physicochemical conditions of the regional crustal rocks. The characteristics of seismic activity are the external manifestation of the physical action mechanism. The statistical characteristics of observational data often reflect the physical and chemical conditions of the regional crustal rocks. Geodetic strain rate has been used in the past as a detection indicator for major earthquakes [46] or as an indicator factor for post-earthquake crustal deformation. The GPS velocity field reflects the kinematic characteristics of tectonic activities, while the strain rate field indicates the pattern and intensity of deformation, to some extent reflecting the characteristics of the tectonic stress field. With the gradual improvement of observation technologies, the current Crustal Movement Observation Network of China consists of 260 reference stations and 2,000 regional stations [47], initially achieving the dynamic monitoring of the first- and second-order tectonic blocks and major active fault zones in the Chinese mainland. In areas where the distribution of GPS points is uneven, the strain rate calculated from the GPS velocity field can better describe the characteristics of tectonic deformation. We utilized the calculation results of the crustal movement velocity field in study area, which is the densest to date obtained by Wang and Shen [48], to explore the correlation with seismicity parameters in study area.

**Fig 10:**
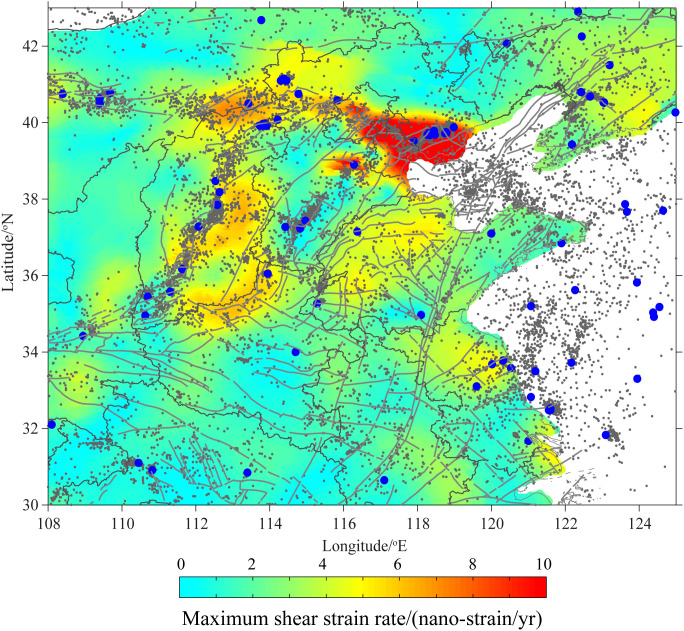
The distributive relationship between the maximum shear strain and seismic activity.

From the perspective of the spatial distribution of strain rate in Fig 10, the strain rate in the epicentral area of the Tangshan earthquake remains high even 30–40 years after the mainshock. This is used as evidence for the existence of a long-term aftershock sequence in the local area [49]. Relatively high shear strain rates basically correspond to the areas where large earthquakes occur, and the current background seismicity level is relatively high. While the correlation between geodetic strain rate and seismic activity is weak within the study area. It is found that there is a certain correlation between seismicity and strain rate. For example, in the Zhangjiakou-Bohai seismic belt, there is a relatively low *b*-value and a high seismic occurrence rate, and at the same time, the strain rate is relatively high. Especially in the Tangshan area with a high background occurrence rate, the maximum strain rate is also relatively large. Similar characteristics also exist in the middle and southern sections of the Shanxi seismic belt.

The *b*-value decreases as the shear strain rate increases. With the intensification of shear deformation, the stress level in the area rises, resulting in a decrease in the *b*-value. The major active tectonic belts, including the Zhangjiakou-Bohai seismic belt and the Shanxi seismic belt, show a correlation among relatively high background seismic activity, high crustal strain rate, and strong earthquakes. However, this correlation is relatively poor within the study area, highlighting the complexity of intraplate earthquakes. Overall, the correlation between strain rate and seismicity in study area is not as strong as that at plate boundaries (such as Southern California) [46]. This is basically consistent with previous research results, indicating that there is a weak or non-existent correlation between geodetic strain rate and seismicity in continental interior regions [50,51].

## Discussion

### Importance of background seismicity in hazard analysis

The background seismic activity obtained through declustering serves as a crucial foundation for calculating seismic activity parameters, conducting seismic probabilistic hazard analysis, and performing disaster assessments [52]. The declustered earthquake catalog (assuming it contains only independent background earthquakes) is typically used as an input for seismic hazard assessment [53,54]. However, as demonstrated in this study, differentiating aftershocks from background earthquakes can be challenging. Gardner and Knopoff [39] and Reasenberg [40] are two commonly used declustering algorithms that follow the stationary Poisson distribution for background seismic activity. Nevertheless, they often lead to the confusion that most earthquakes are deleted in the short term after significant earthquakes. In contrast, the stochastic declustering method not only satisfies the Poisson distribution but also preserves a large number of earthquakes [3]. Moreover, it is one of the rare methods that do not alter the significant statistical characteristics of earthquakes [55].

### Challenges and limitations of declustering algorithms

Due to the spatio-temporal complexity of real seismic activities, declustering algorithms merely provide a rough description and identification of earthquake sequences, and they are constantly being refined and developed. The applicability of declustering algorithms can also be influenced by differences in tectonic environments. Most declustering methods rely on changes in statistical characteristics within the catalog, and the separation of aftershock and background seismic activities in statistical models has no relation to the underlying physical mechanisms. As Zaliapin and Ben-Zion [56] put it, “We don’t even know if natural seismic activity really operates in terms of these concepts (background, clustering, etc.).”

The complex intraplate tectonic activities make the calculation of background seismicity rate more difficult. This is because earthquake records in these areas are often incomplete, and the recording duration is insufficient to reflect the long-term and slow tectonic loading. Therefore, we calculated the background seismicity under different cut-off magnitudes and compared the impacts they brought. The background seismic activities in study area determined in this study are mainly distributed along major fault zones in space and basically exhibit a certain stable distribution pattern in time. Xiong et al. [20] calculated the total seismic spatial occurrence rate and the background seismic spatial occurrence rate in study area using four different spatio-temporal ETAS models. Through comparative analysis and simulation experiments, it was shown that the spatial patterns of earthquake occurrence given by these methods have similar characteristics. Consistent with the conclusions in this paper, a high seismic activity rate area are mainly distributed along seismic belts, which are also areas with a relatively high background seismicity rate.

### Intraplate seismicity and its implications

In intraplate regions, the challenges are even greater because the slow tectonic loading is shared among complex fault systems and fault networks. In the long term, intraplate earthquakes tend to cluster in time and migrate in space [49,51]. In study area, our analysis of earthquake catalogs since 1980 shows that the Zhangjiakou-Bohai seismic belt has a relatively high background seismic activity rate. These regions also exhibit relatively high crustal strain rates and are concentrated with strong earthquakes. However, the correlation between background seismic activity, strain rate, and strong earthquakes is not clear, within the study area. This is in good agreement with the research by Chen et al. [57] on the relationship between background seismic activity and strain rate based on the nearest neighbor method. In the more stable and less active North American Craton, Kreemer et al. [50] found no correlation between seismic activity and plate-scale strain rate.

We analyzed the seismic hazard in study area solely from the perspective of seismic activity. However, non-seismic activity data can assist us in constructing models with higher forecasting performance. In the future, better results can be achieved when more physical and geological data, such as GPS displacements, are incorporated. When conducting statistical modeling using modern earthquake catalogs, it is necessary to compare and analyze the methods. Further comprehensive regional seismic hazard analyses may require the integration of fault data [16,58], GPS data [46], and other information [58–60].

## Conclusions

The selection of the cut-off magnitude can lead to certain differences in the declustering results. Specifically, as the cut-off magnitude increases, the declustering rate shows a downward trend. Moreover, due to the reduction in the number of samples, the declustering rate fluctuates significantly. The variation trends under different cut-off magnitudes are generally consistent. There is no significant correlation between the seismicity parameters (*a*, *b*) of all earthquakes, background earthquakes, clustered earthquakes and the selection of the background probability and the cut-off magnitude threshold. The stochastic declustering method has a relatively low dependence on the magnitude and will not significantly change the spatio-temporal statistical characteristics of seismicity parameters.

Different cut-off magnitudes have varying degrees of influence on the spatial distribution of the *b*-value. However, the overall low *b*-value regions are consistent. That is, in the western part of the northern margin of the Ordos, the intersection area of the Zhangjiakou-Bohai seismic belt and the Tanlu seismic belt, there are relatively large areas of low *b*-value anomalies. The stress state in these areas is at a relatively high level, indicating a relatively high risk of strong earthquakes in the future. The areas with abnormal low *b*-values in study area are highly consistent with the evolution of the current Coulomb stress. A profound understanding of the seismic activity state based on the spatio-temporal ETAS model can facilitate the exploration of the laws and characteristics of seismic activities. This, in turn, enhances the objectivity and reliability of earthquake forecasting, thereby providing strong support for reducing the losses caused by earthquake disasters.

Under different cut-off magnitudes, the background earthquake occurrence rate exhibits similar distribution characteristics, mainly along fault zones. However, as the cut-off magnitude increases and the number of samples decreases, the background earthquake occurrence rate shows an obvious downward trend, and the areas with high background occurrence rates tend to shrink. Considering the spatial distribution of the clustering rate, the Zhangjiakou-Bohai seismic belt, the middle and southern sections of the Shanxi seismic belt, the middle and northern sections of the Tanlu seismic belt, and the southwestern Yellow Sea are areas that require key attention in the future. Additionally, most significant strong earthquakes are distributed along seismic belts, this is also an important factor contributing to the higher background seismicity rates in these belts. High strain rate regions are predominantly located within or near seismic belts. Therefore, there exists a certain correlation among background seismicity, strain rates, and strong earthquakes in some major seismic belts. For example, the Zhangjiakou-Bohai seismic belt demonstrates a significant correlation among relatively high background seismicity, high crustal strain rate, and strong earthquakes. In contrast, within the study area, this correlation is relatively weak, highlighting the complexity of intraplate earthquakes.

The seismic hazard analysis conducted under different cut-off magnitudes provides a multi-layered understanding of regional seismicity. Lower cut-off magnitudes reveal the cumulative effects of smaller earthquakes, which are critical for assessing long-term infrastructure stability. Higher cut-off magnitudes highlight the probability and potential impact of large earthquakes, offering essential insights for disaster preparedness and mitigation. Based on the analysis results of the background occurrence rate of the spatio-temporal ETAS model under different magnitude cut-off, the seismic hazard of different regions in study area can be evaluated more accurately. This provides a scientific basis for urban planning, engineering construction, etc., enabling the adoption of corresponding seismic fortification measures. Future research should focus on optimizing cut-off magnitude selection and integrating multi-magnitude analysis results to enhance the applicability and accuracy of seismic risk assessments.

Copyright: ©2024 Bi et al. This is an open access article distributed under the terms of the Creative Commons Attribution License, which permits unrestricted use, distribution, and reproduction in any medium, provided the original author and source are credited.

## Supporting information

S1 Data S1 Table. Data of Figs 2 and 3. The distribution map of seismic activities in North China and the Magnitude-frequency distribution and magnitude completeness analysis results in North China. S2 Table. Data of Fig 4. Declustering results under different cut-off magnitudes. S3 Table. Data of Fig 5. The relationship between threshold setting and seismicity parameters. S4 Table. Data of Fig 6. Distribution characteristics of *b*-values for all earthquakes and different cut-off magnitudes. S5 Table. Data, S6 Table. Data and S7 Table. Data of Fig 7. Variation characteristics of the *b*-value in the Zhangjiakou-Bohai seismic belt. S8 Table. Data of Fig 8. Distribution characteristics of background seismicity under different cut-off magnitude. S9 Table. Data of Fig 9. The spatial distribution of the clustering rate under different cut-off magnitude. S10 Table. Data of Fig 10. The distributive relationship between the maximum shear strain and seismic activity.
(RAR)
